# Complementary Treatment in Chronic Pelvic Pain Syndrome: A Case Report Study

**DOI:** 10.5812/ircmj.13681

**Published:** 2014-04-05

**Authors:** Seied Amirhossein Latifi, Bagher Minaiee, Mohammad Kamalinejad, Esmaeil Nazem, Shahram Gooran

**Affiliations:** 1 School of Traditional Medicine, Tehran University of Medical Sciences, Tehran, IR Iran; 2 Department of Histology, School of Medicine, Tehran University of Medical Sciences, Tehran, IR Iran; 3 Department of Pharmacognosy, School of Pharmacy, Shahid Beheshti University, Tehran, IR Iran; 4 Department of Urology, Sina Hospital, Tehran University of Medical Sciences, Tehran, IR Iran

**Keywords:** Medicine, Traditional, Pelvic Pain, Treatment

## Abstract

**Introduction:**

The use of traditional medicine has been emerged in the treatment of BPS (bladder pain syndrome) due to its high prevalence and expenses and its insufficient treatment by conventional therapies. Iranian traditional medicine has discussed such diseases. Considering the signs and symptoms of BPS and “reeh”, the proposed mechanism of flatulency as casualty of recurrent circulating pains seems to be a proper diagnose. So, as a preliminary study the authors administered Horse Mint as one of effective traditional herbs on flatulent pain in a patient with BPS.

**Case Presentation:**

A 60-year-old female was referred with the diagnosis of BPS. Six clinical visits with 2-week intervals were performed for patient, and the NIH-ICSI (National Institutes of Health Interstitial Cystitis Symptom Index) was completed, which was used as a pretreatment symptom quantifier and post-treatment outcome tool. Horse mint (*Mentha longifolia*) was prescribed twice a day for 12 weeks.

**Discussion:**

Clinical visits showed alleviation of signs, symptoms, and changes in the patient's NIH-ICSI score, suggesting further studies on this field.

## 1. Introduction

According to the final consensus of the European Society for the Study of Interstitial Cystitis (ESSIC), persistent occurrence of chronic pelvic pain syndrome (CPPS) symptoms for more than six months accompanied with pain and discomfort in the bladder area and at least one of the urinary symptoms such as urinary urgency and frequency is considered as Bladder Pain Syndrome (BPS) (1). Indirect costs of treatment for this syndrome have been estimated to be higher than the average value of other chronic pains (2). Additionally, higher prevalence of mental health impairment including depression, stress, panic attacks and sexual dysfunction among these patients has had deep effects on patients and their families, suggesting that control of symptoms of this syndrome should be taken more seriously (3). In spite of numerous etiological hypotheses, CPPS is still unknown and has no definitive treatment.

Relying on evidence-based medicine, clinical guidelines for chronic pelvic pains have been prepared with a multidisciplinary approach. EAU guidelines on CPP have suggested the use of complementary treatment as a supportive second-line therapy in the diagnosis and treatment algorithms of bladder pain (4, 5). The positive effects of many complementary medicine treatment methods such as massage therapy, acupuncture, biofeedback, hyperthermia, and herbal medicine have been confirmed in various studies (6). This study aimed to find a treatment method based on the results of diagnostic approaches of Iranian traditional medicine. This approach was examined on a patient with the diagnosis of BPS/IC in the Urology clinic of . *Mentha longifolia* was prescribed for the patient and the results were evaluated.

## 2. Case Presentation

A 60-year-old female housewife was admitted to the Urology Clinic of Sina Hospital, Tehran (capital of Iran) eight months before admission (winter of 2013) with pain in the suprapubic area (Table 1). The patient had been treated by antibiotic therapy since eight months ago following intense suprapubic pains accompanied by dysuria and frequency symptoms and had been diagnosed as simple cystitis. In spite of partial recovery and continuation of long-term treatment with antibiotics, non-steroid anti-inflammatory drugs, sedative drugs and alpha-blocker, the symptoms were recurred with severe and mild periods. The pain was persistent and spread towards the perineal area. Along with the pain, the patient had frequency, nocturia and dysuria, which resulted in inability to pass urine completely. The pain intensified when the bladder was full and the patient also had occasional flank pain on both sides. In abdominal physical examination, slight tenderness in the suprapubic area was found, but the rest of examination had normal findings. In paraclinical tests, urine culture test had normal results, and urine analysis showed normal findings regarding the values of white blood cell, red blood cells, sugar and protein. Urine cytology had negative results regarding malignancy. Abdomen and pelvic sonography and the liver and kidney function test had normal results.

**Table 1.** Patient Characteristics ^a^

	Characteristics
**Demographic Data**	Age, Y = 60, job = housewife, education = low, BMI = 25-29.9, smoking = no, parity = yes
**Symptoms**	suprapubic pain, dysuria, frequency, nocturia, incomplete voiding
**Duration of symptom, m**	> 8
**Previous treatment**	tamsulosin, ofloxacin, baclofen, nitrofurantoin, fluoxetine
**Diagnostic Testing**	cystoscopy with biopsy, sonography, urine cytology , D&C
**Comorbid Condition**	Osteoarthritis, constipation, hemorrhoid, diabetes mellitus, chronic low back pain

^a^ Abbreviation: BMI, body mass index.

In cystoscopy report, the size of bladder was slightly decreased and there was a mild inflammation of the epithelial lining of the bladder. No tumoral lesion or glomerulation was observed. Bladder biopsy and hydro distention were performed. Bladder biopsy had negative results for malignancy, but chronic inflammation was reported. The results of the examination of the genitalia system in physical examination and dilation and curettage (D&C) had normal findings.

### 2.1. Past Medical History

The patient reported diabetes mellitus type 2 for three years and used metformin daily. FBS was 120 mg/mL. The digestive problems of the patient were a persistent bloat for many years accompanied by pain in the epigastrium and the umbilical area, constipation, and hemorrhoid without bleeding. The musculoskeletal problems of the patient were chronic osteoarthritis in both knees and chronic low back pain, which had reduced the physical activities of the patient to a sedentary life. This patient was chosen because of presence of both symptoms of BPS and “reeh”. Positive “reeh” signs and symptoms included:

Abdominal distention with boring quality, urinary symptoms, and non-localized and shifting pains in the abdomen with sudden onset relived easily, absence of feeling heaviness in the pain position, severity of the pain with consumption of flatulent foods like peas.

### 2.2. Intervention

ICSI Score, numeric pain rating scale (NPRS) (which scales the pain from one to ten) and Impact of symptoms/quality of life (QOL) (a questionnaire with negative scaling of quality of life) score results were recorded in each examination session before starting the treatment (7, 8). ICSI assesses urinary symptom (urgency, frequency and nocturia). These were used as indicators of the symptoms before the treatment and a tool for keeping track of the treatment process. The patient took the infusion of two grams of dried horsemint before lunch and dinner. The dose was very lower than the toxic dosage. New reports of her condition were prepared every 14 days, after either visiting her or making phone calls. Measuring variable was performed by taking history, clinical examination and using the questionnaires by supervising an expert urologist.

## 3. Discussion

After three months of follow-up, progressive healing was revealed in pain, quality of life, signs and symptoms such as frequency, nocturia and dysuria. Symptoms of “reeh” showed a dramatic healing. No side effect was reported. The results are presented in Table 2.

**Table 2.** Pre and Post Therapy ICSI Score, NPRS and QOL Impact Score ^a^

Measure	Pre therapy	Post therapy					
		Visit 1	Visit 2	Visit 3	Visit 4	Visit 5	Visit 6
**ICSI-symptoms-score (9)**	11	9	9	7	6	6	3
**NPRS-score (0-10 scale)**	8	6	5	4	4	0	0
**Impact of symptom/QOL –score (10)**	9	7	7	5	5	3	3
**Total score**	28	22	21	16	15	10	6

^a^ Abbreviations: ICSI, interstitial cystitis symptom index; NPRS, numeric pain rating scale; QOL, quality of life.

Avicenna had discussed bladder illnesses and methods for identifying distinctive causes in his Canon of Medicine using his own special terminology. The Canon of Medicine is a unique source book for describing the semiology of these diseases (11). Examining the condition of the aforementioned patient from the viewpoint of traditional medicine showed that her non-localized pains in the flanks, back, around the umbilicus, and above the bladder along with the simultaneous presence of urinary symptoms and digestive and musculoskeletal problems could all be under a same common etiology. The distinctive feature of the traditional approach towards this case in comparison with the modern approach is that it associates all of the above symptoms which have unknown causes with a common cause named “reeh” (9). It is a current flow in the body, which acts like “wind” as its counterpart in the nature and plays expanding role in pelvic organs' ducts to facilitate execratory functions, such as urination, defecation and erection/ejaculation in physiologic condition. The problem is in fact a disorder in the excretion process of the kidney and the bladder which has resulted in distensile flatulent pain. The pain with mechanism of “reeh” is reported to be distending, stabbing or boring in quality. It is a non-localized shifting pain without sensation of heaviness by the patient. It has a sudden and severe onset which is yet easy to relieve at certain times, especially in good digestive conditions. Based on this mechanism traditional medicine can explain the high correlation between digestive problems and sexual dysfunction in these patients. *Mentha longifolia*, with the common name “horse mint” is a known herbal medicine with anti-spasm properties and also anti-microbial effects against gram-positive and gram-negative bacteria (10). Additionally, the anti-oxidant properties of its essence and extracts have been in focus of attention in recent studies (12). Avicenna has referred to this plant as “foodanj” in his Canon of Medicine and has mentioned its effects in treatment of urinary symptoms of bladder problems, as well as a wide range of therapeutic effects on lung diseases such as bronchitis, gynecological illnesses such as secondary amenorrhea, and liver diseases such as jaundice (9). Horse mint could gradually cure the patient because of its anti-flatulent properties. Week points of our study was its low amount of data and probable instrumental error caused by translation of questionnaires from English to Persian language which should be reinforced and considered by further case reports and future clinical trials. Presence of both signs and symptoms of “reeh” (in traditional medicine view) and BPS (in conventional medicine view) in this patient was a strong point of study, which should be considered in the future studies.

The results of this study and considerable recovery of the patient for the symptoms and quality of life could be a starting point for further evidence based studies in this area.
